# Web-Based Mindfulness-Based Cognitive Therapy for Adults With a History of Depression: Protocol for a Randomized Controlled Trial

**DOI:** 10.2196/53966

**Published:** 2024-06-18

**Authors:** Mohammad Hooshmand Zaferanieh, Lu Shi, Meenu Jindal, Liwei Chen, Lingling Zhang, Snehal Lopes, Karyn Jones, Yucheng Wang, Kinsey Meggett, Cari Beth Walker, Grace Falgoust, Heidi Zinzow

**Affiliations:** 1 University of South Carolina School of Medicine Greenville Greenville, SC United States; 2 Department of Health Sciences College of Health Professions Pace University New York, NY United States; 3 Department of Internal Medicine Prisma Health Greenville, SC United States; 4 Fielding School of Public Health University of California Los Angeles Los Angeles, CA United States; 5 Manning College of Nursing and Health Sciences University of Massachusetts Boston Boston, MA United States; 6 College of Behavioral, Social and Health Sciences Clemson University Clemson, SC United States

**Keywords:** mindfulness-based cognitive therapy, MBCT, mindfulness-based interventions, depression, depressive symptoms, virtual delivery, mindfulness, mental health, depressive, distress, stress, remote, randomized, controlled trial, controlled trials, RCT, psychotherapy, cognitive therapy

## Abstract

**Background:**

Depression poses a major threat to public health with an increasing prevalence in the United States. Mindfulness-based interventions, such as mindfulness-based cognitive therapy (MBCT), are effective methods for managing depression symptoms and may help fortify existing efforts to address the current disease burden. The in-person group format of MBCT, however, incurs barriers to care such as expenses, childcare needs, and transportation issues. Alternate delivery modalities such as MBCT delivered via the web can be investigated for their capacity to overcome these barriers and still reduce symptoms of depression with adequate feasibility and efficacy.

**Objective:**

This study protocol aims to examine the feasibility and efficacy of MBCT delivered via the web for the treatment of depression.

**Methods:**

To attain study aims, 2 phases will be implemented using a waitlist control design. A total of 128 eligible participants will be randomized into either an 8-week MBCT intervention group plus treatment as usual (MBCT + TAU; group 1) or an 8-week waitlist control group (group 2). In phase I (8 weeks), group 1 will complete the intervention and group 2 will proceed with TAU. In phase II (8 weeks), group 2 will complete the intervention and group 1 will continue with TAU until reaching an 8-week follow-up. TAU may consist of receiving psychotherapy, pharmacotherapy, or combined treatment. Data collection will be completed at baseline, 8 weeks (postintervention for group 1 and preintervention for group 2), and 16 weeks (follow-up for group 1, postintervention for group 2). The primary outcomes will include (1) current, residual, or chronic depression symptoms and (2) psychiatric distress. Secondary outcomes will include perceived stress and facets of mindfulness. The feasibility will be measured by assessing protocol adherence, retention, attendance, and engagement. Finally, the extent of mindfulness self-practice and executive functioning skills will be assessed as mediators of intervention outcomes.

**Results:**

This study began screening and recruitment in December 2022. Data collection from the first cohort occurred in January 2023. By November 2023, a total of 30 participants were enrolled out of 224 who received screening. Data analysis began in February 2024, with an approximate publication of results by August 2024. Institutional review board approval took place on September 11, 2019.

**Conclusions:**

This trial will contribute to examining mindfulness-based interventions, delivered via the web, for improving current, residual, or chronic depression symptoms. It will (1) address the feasibility of MBCT delivered via the web; (2) contribute evidence regarding MBCT’s efficacy in reducing depression symptoms and psychiatric distress; and (3) assess the impact of MBCT on several important secondary outcomes. Findings from this study will develop the understanding of the causal pathways between MBCT delivered via the web and depression symptoms further, elucidating the potential for future larger-scale designs.

**Trial Registration:**

ClinicalTrials.gov NCT05347719; https://www.clinicaltrials.gov/ct2/show/NCT05347719

**International Registered Report Identifier (IRRID):**

DERR1-10.2196/53966

## Introduction

Major depressive disorder (MDD) is a serious threat to health in the United States and other middle and high-income countries [1], with a lifetime prevalence rate close to 20% [2]. Those with MDD have both the symptoms of depression and impairments in quality of life [3]. Moreover, MDD can place economic burdens on families and society at large [4]. The average annual costs per depression case can range from US $1000 to $2500 for direct costs, from US $2000 to $3700 for morbidity costs, and from US $200 to $400 for mortality costs [5].

To address chronic challenges to community health such as depression, public health stakeholders seek out programs that can be easily delivered in a web-based setting [6]. Mindfulness-based interventions (MBIs) have been increasingly recognized as efficacious [7] and cost-effective [8] in addressing chronic issues in mental health and substance misuse. Originating from Eastern meditation and yoga traditions, MBIs are designed to foster greater awareness of present-moment experiences [9]. Recent evaluations found that MBIs with a standardized protocol saved health care expenditures for patients with various physical or mental health issues [10,11]. With their consistent benefit on mental health, substance misuse, and physical fitness outcomes [7,12-14], MBIs represent a promising strategy for community organizations dedicated to improving the health of their residents.

Particularly, mindfulness-based cognitive therapy (MBCT) has been shown to be an effective method of reducing symptoms of depression [15-20]. MBCT has also been shown to decrease depression symptoms in individuals with chronic and treatment-resistant depression, as well as residual symptoms of recurrent depression [21-23]. MBCT is an 8-session group intervention program designed to increase awareness of internal reactions that may lead to symptoms of depression. These internal processes may include depression-related rumination and persistent negative thoughts. By equipping individuals with techniques to detach from these dysfunctional cognitive patterns, MBCT has the potential to redirect attention to the current, present-moment experiences [20]. Additionally, MBCT has been associated with several mechanisms of change purported to impact depressive symptoms, including increases in mindfulness and self-compassion in addition to decreases in cognitive reactivity to negative, ruminative thinking [24-26].

Further, 1 challenge surrounding in-person MBCT is the financial barrier that may prevent at-risk populations from accessing the care that they need. The average cost for an 8-session program ranges from US $100 to $250 per session, making it inaccessible for individuals with limited financial resources. What is particularly striking is that individuals with lower incomes are at an increased risk for depression [27] as well as other mental health disorders [28]. This points to the importance of investigating what role MBCT delivered via the web may play for at-risk populations.

A benefit commonly associated with web-based adaptation is decreased cost [29-32]. A web-based format can reduce or eliminate expenses like facilities, food, and traveling fees [33]. Decreased costs enable the interventions to be accessible to a wider range of participants, companies, and researchers at a lower price, allowing for broader dissemination and increased study size. Technology-based delivery also addresses many issues pertinent to administering interventions, such as reaching groups who live in remote locations or who lack adequate transportation [30,34,35]. Web-based platforms enable participants to complete evidence-based intervention sessions in the privacy of their own homes, potentially protecting anonymity and reducing the stigma associated with taking part in a mental health intervention [36,37]. Finally, technology-based administration also eliminates the need to travel to sessions or arrange for childcare, increasing convenience and reducing time commitment for participants [38,39].

However, despite the numerous benefits associated with formats delivered via the web, there are challenges associated with adapting interventions originally designed for in-person delivery. Further, 1 reason for concern is that adaptations that remove fidelity, or key elements of an intervention, harm the efficacy of the intervention [40]. In addition to the potential loss of fidelity of the MBCT program, MBCT delivered via the web also presents challenges in retaining participant attention, maintaining the quality of group discussion, and combating “zoom fatigue.”

Our study will take steps to address these concerns, testing the feasibility of the web-based, or online-delivery model of MBCT. Such steps will include making recommendations to participants to keep their video on during the intervention session, remaining in a quiet area, making prior arrangements to minimize interruptions, regular check-ins during the session to stimulate participant response to exercises and questions, using headphones if available, having water and a blanket nearby in case needed, and finally, ensuring that a device charger is at hand. In addition to addressing feasibility concerns, we aim to contribute to the evidence base regarding MBCT’s efficacy in reducing depressive symptoms. Finally, we hope to add to the understanding of the causal mechanisms and factors that moderate treatment outcomes of MBCT for depressive symptoms.

## Methods

### Study Design

Our study plans to examine how MBCT delivered via the web may affect primary and secondary outcomes for individuals with a history of depression. It will also assess mediators, moderators, and the sustainability of the intervention. The following hypotheses will be assessed among program participants in the intervention group as compared to a waitlist control group, and within groups compared from pre- to postintervention.

- The MBCT intervention group will show a greater reduction in depressive symptoms from TI to TII, compared to the waitlist group.
- The MBCT intervention group will show a greater reduction in psychiatric distress symptoms from TI to TII compared to the waitlist group.
- The MBCT intervention group will sustain gains through the 8-week follow-up period at TIII.
- The MBCT intervention will improve secondary outcomes, such as:
  perceived stress
  facets of mindfulness
- The MBCT intervention will improve the skills associated with mechanisms of change for individuals with a history of depression, and these skills will act as mediators between MBCT and primary and secondary outcomes:
  extent of mindfulness self-practice
  executive functioning skills
- The format delivered via the web will meet feasibility aims measured by adherence, retention, attendance, and engagement.

### Trial Design

**Figure 1:**
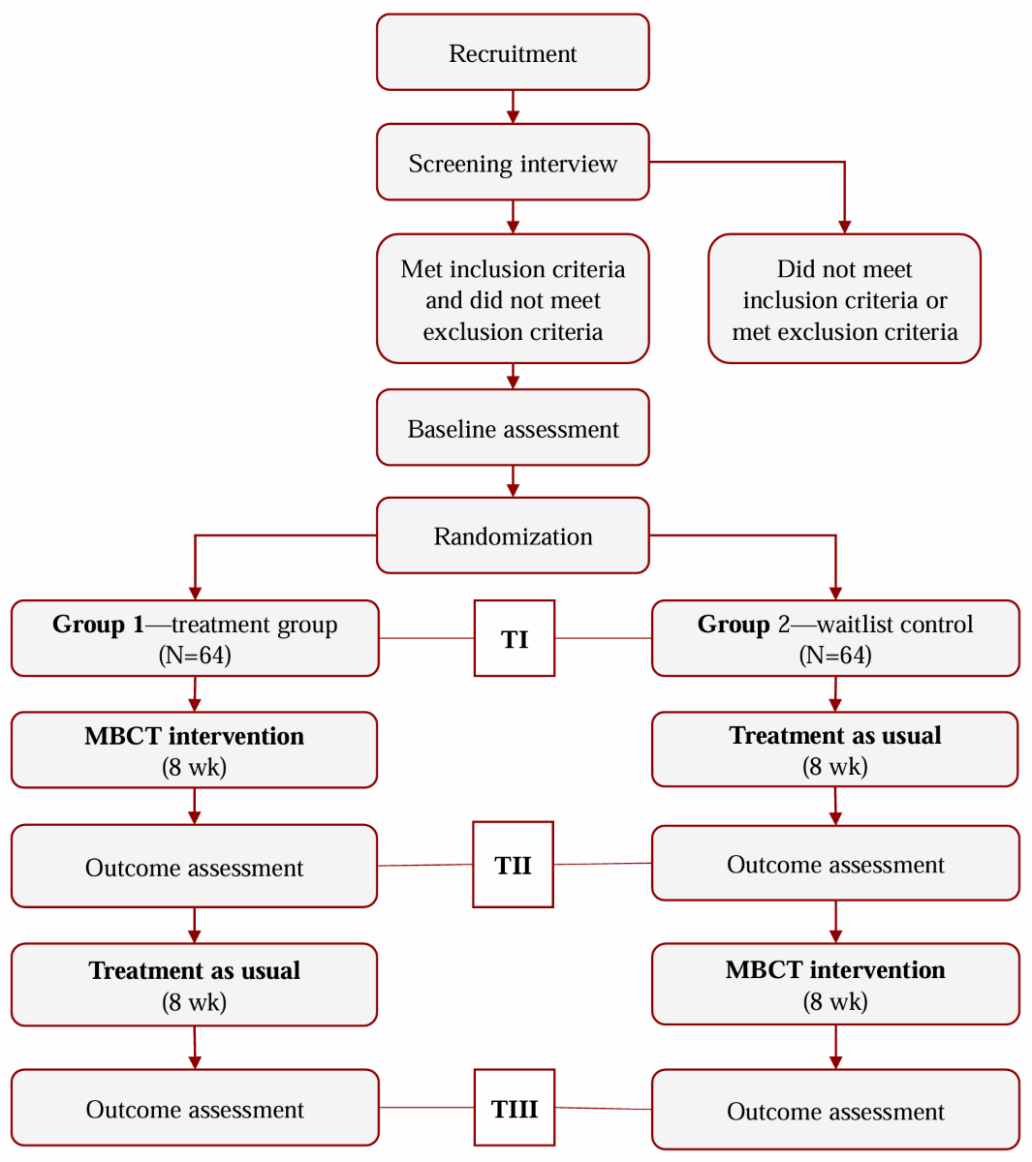
Study flow. MBCT: mindfulness-based cognitive therapy; T: timepoint.

Using a waitlist control design, this trial will randomize 128 eligible participants into 2 groups. In the first phase lasting 8 weeks, group 1 will partake in the MBCT intervention plus treatment as usual (MBCT + TAU) while group 2 will remain as waitlist control following TAU. In the second phase lasting 8 weeks, group 2 will begin completing the MBCT intervention, and group 1 will enter a follow-up phase, continuing with TAU. TAU will be comprised of psychotherapy, pharmacotherapy, or a combination of both. Data collection will be completed at baseline, 8 weeks (postintervention for group 1 and preintervention for group 2), and 16 weeks (follow-up for group 1 and postintervention for group 2). In this manner, our study design will ensure that both trial arms are treated fairly, and that group 1 can be followed up for 8 weeks to assess the sustainability of the intervention. Figure 1 outlines the flow of this study. The registration of this trial took place on April 22, 2022.

### Participants and Procedure

#### Setting

This study will be led by Prisma Healthcare System and Clemson University, in collaboration with the Prisma Health-Upstate Internal Medicine Clinic. The clinic is a branch of the Prisma Health System that provides community-based primary care and acts as an academic tertiary care site. As part of their goal to address the chronic disease burden, health care providers at the clinic screen approximately 700 patients monthly for depression.

#### Eligibility Criteria

Participants in this study must (1) be aged 18 years at a minimum, (2) speak English fluently, (3) have sixth grade and above literacy, (4) be willing to share contact information (home address, phone number, or email), (5) have no current mindfulness practice, (6) be Greenville residents or persons who are patients of the partnering organizations in our study, and (7) have current or a lifetime history of any major depressive episode. Study exclusion criteria include (1) current psychosis, dementia, moderate to severe traumatic brain injury, pregnancy, or active suicidality; (2) persistent antisocial behavior; (3) persistent self-injury requiring clinical management; (4) an acute episode of a substance use disorder episode (met 2 or more substance use disorders criteria in the past 2 weeks); (5) previously completed or currently attending an MBI; and (6) are currently younger than 18 years, are non–English speakers, or have a literacy lower than sixth grade.

#### Recruitment and Screening Procedure

Participants will be recruited from the Prisma Health-Upstate Internal Medicine Clinic who currently have or have had a lifetime history of any major depressive episode. Health care providers at the clinic will identify patients using the *ICD-11* (*International Classification of Diseases, 11th Revision*) codes [41] from the patients’ electronic medical records. Researchers will contact eligible participants and ask for verbal consent before participation in an in-person screening interview with a member of the research team. At this time, the Patient Health Questionnaire (PHQ-9) [42] is to ascertain any current depression symptoms. PHQ-9 scores of 5, 10, 15, and 20 will represent mild, moderate, moderately severe, and severe depression, respectively. If no depression symptoms are currently present, this will also be noted. Participants who meet eligibility criteria will be asked to provide written informed consent before enrolling in this study. Recruitment began in December 2022.

#### Intervention Procedures

The intervention will be delivered by 3 Licensed Clinical Social Workers who have previously undergone MBCT teacher training and become certified instructors. The intervention will consist of 8 weekly 2-hour group therapy sessions. All sessions will be delivered to a group of up to 12 participants and will take place over Zoom (Zoom Video Communications, Inc). The minimum number of participants needed to proceed with each intervention program is 7 individuals. Certified instructors will follow the standardized MBCT protocol developed by Segal et al [43] (see Textbox 1 for the outline of each session).

**Mindfulness-based cognitive therapy program outline per Segal et al [43].**

Session 1: Awareness and automatic pilot

Session 2: Living in our heads

Session 3: Gathering the scattered mind

Session 4: Recognizing aversion

Session 5: Allowing or letting be

Session 6: Thoughts are not facts

Session 7: How can I best take care of myself?

Session 8: Maintaining and extending new learning

MBCT integrates training in mindfulness meditation and cognitive-behavioral methods. It teaches skills designed to increase awareness of internal reactions that trigger depressive symptoms, such as depression-related rumination and negative thoughts, and provides techniques to detach from these dysfunctional cognitive processes [19]. In the first half of the program, participants will focus on learning the principal tenets of mindfulness. They will explore central themes about the tendencies of the mind to habitually engage with thoughts, which produces an array of feelings and emotions. In the latter half of the program, participants will begin the practice of becoming aware of thoughts as they arise, thereby witnessing habitual patterns that may lead to unpleasant feelings or emotions [43]. Outside of the sessions, participants will be assigned daily homework exercises and given handouts as well as audio recordings to use in their mindfulness practice. Participants who miss a session will be contacted by the research team to determine if they would benefit from medical or psychiatric assistance and to assess any barriers to upcoming sessions.

#### Study Fidelity

The intervention’s fidelity will be evaluated using the MBCT Assessment Scale [44]. Weekly session audio recordings will be archived for access by 2 members of the research team who shall conduct the assessment. They will each use the MBCT Assessment Scale to code a random selection of half of the sessions. To achieve reliability, they will conduct 10 practice assessments which will be vetted by the research team. To prevent drift, frequent recalibration meetings will be scheduled. Additionally, group facilitators will convene weekly in the presence of the licensed clinical psychologist for supervision.

#### Study Withdrawal

Participants will not be permitted to continue in this study if uncooperative behavior is displayed threatening group cohesion, or if participants undergo severe psychological or medical symptoms that warrant professional treatment. If a participant does not attend an intervention session, the research team will follow up with that individual. A record will be maintained of contact information for participants’ usual providers and other emergency contact persons, and these individuals will be contacted if the participant cannot be reached. If following 5 weekly attempts to reach a participant no response is obtained, the participant will be considered lost to follow-up. Those who wish to voluntarily discontinue participation in this study will be asked the reasoning behind their decision.

### Assessment Procedures

**Figure 2:**
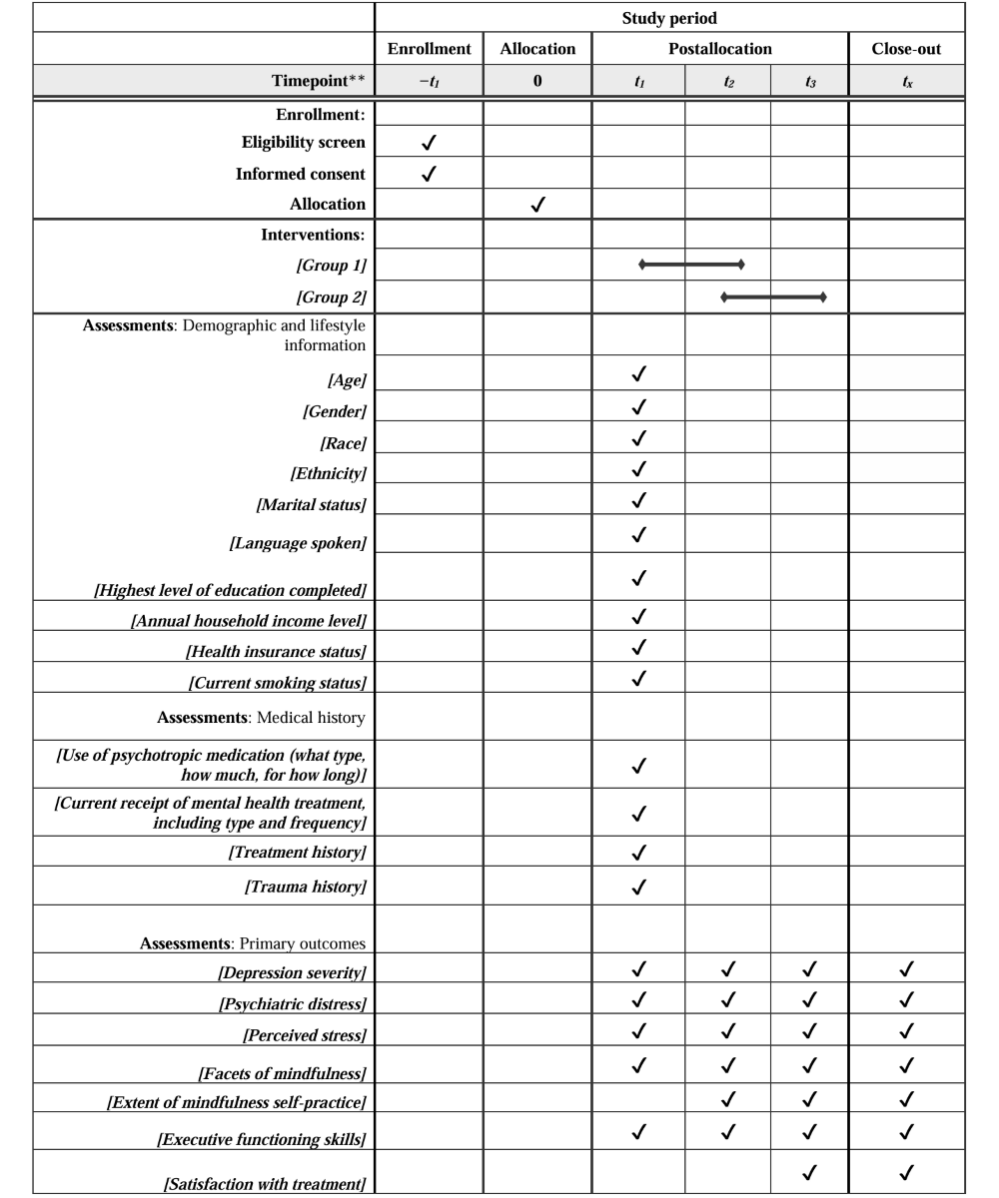
Schedule of enrollment, interventions, and assessments.

This study will conduct data collection at 3 different time points: TI—baseline, TII—postintervention for group 1 and preintervention for group 2 (8 weeks), and TIII—follow-up for group 1 and postintervention for group 2 (16 weeks). Participants will complete web-based self-reported surveys administered via Qualtrics (Qualtrics). If the participant prefers, paper surveys will be administered. Figure 2 lists study measures and assessment points.

### Primary Outcome Measures

#### Depression Symptoms

Depression symptoms will be assessed with the PHQ-9 [42].

#### Psychiatric Distress

Psychiatric distress will be measured using the depression and anxiety subscales of the Brief Symptom Inventory [45].

### Secondary Outcome Measures

#### Perceived Stress

Using the Perceived Stress Scale-4 [46], perceived stress will be investigated.

#### Facets of Mindfulness

The Five Facet Mindfulness Questionnaire [47] will be used to assess self-reported mindfulness. In total, the 5 subscales include (1) nonreactivity, (2) observing, (3) describing, (4) nonjudgment, and (5) acting with awareness.

### Possible Mediators

#### Extent of Mindfulness Self-Practice

Participants will be asked the following questions: “Besides the sessions you may have attended as part of this study, on your own, ‘Did you engage in mindfulness meditation or other mindfulness practices in the past two months (8 weeks)?’ ‘How many days per week did you engage in mindfulness meditation or other mindfulness practices?’ ‘How long in minutes did you meditate per session of mindfulness meditation?’ ‘Describe your practice of mindfulness (what exercises/activities/techniques did you practice?).’” For quantitative analyses, we will use the variable representing the number of days of mindfulness practice per week.

#### Executive Functioning

The 20-item self-reported Deficits in Executive Functioning Scale, Short Form will be used [48].

### Participant Characteristics and Possible Moderators

#### Demographics

We will assess age, gender, race, ethnicity, marital status, language spoken, educational attainment, employment status, annual household income, health insurance status, and current smoking status.

#### Treatment History

Mental health treatment history will be assessed using the Adult Service Use Schedule [49-51].

#### Trauma History

Adverse life events and trauma history will be assessed with the Adverse Childhood Experiences Questionnaire [52] and Life Events Checklist for the *DSM-5* (*Diagnostic and Statistical Manual of Mental Disorders* [Fifth Edition]) [53].

#### Baseline Depression Symptoms

The PHQ-9 [42] will be used to assess baseline depression symptoms. Participants will be subdivided into mild depression (scores less than or equal to 10) or moderate to severe depression (scores greater than 10) for subgroup comparisons.

### Feasibility Outcomes

#### Retention and Attendance

Retention and attendance will be assessed via the number of sessions attended and the percentage of participants who completed all sessions.

#### Engagement

Engagement will be measured per the items described above that assess the frequency and extent of mindfulness practice.

#### Satisfaction With Treatment

Treatment satisfaction and perceived utility of treatment will be assessed with the Treatment Satisfaction Survey [54] adapted for our study. This version will reflect how participants are satisfied with and respond to the MBCT intervention.

#### Format Delivered via the Web

To determine the feasibility of the web-based format, participants will be asked about strengths, weaknesses, and areas for improvement.

### Sample Size

The total sample size of our trial is expected to be 128 individuals with 64 participants placed into each group. This was estimated by a 2-tailed *t* test using GPower 3.1 (Informer Technologies, Inc) software. Our calculation of sample size depended on (1) the level of significance (α error probability), which was set as .05 based on Ronald A Fisher’s suggestion [55]; (2) the expected effect size, which was set as *d*=0.5, according to Cohen classification that “a medium effect of .5 is visible to the naked eye of a careful observer” [56]; (3) the expected power of this study (1–β error probability), which was set as 0.8 based on Cohen theory [57] because we failed to obtain support from previous similar studies; and (4) the allocation ratio (N2/N1), for which we expect number of participant in treatment group and control group to be the same.

### Randomization

Consenting participants will be randomized 1:1 to either group 1 or group 2 via the randomization function found in Qualtrics. This will be conducted by the principal investigator (PI). Once the number of participants has reached 7-12 individuals for both group 1 and group 2, this study will commence. This will proceed continuously until the expected sample size of 128 individuals is reached, or 4 years have elapsed. The implementation of this process will be overseen by the clinical research team.

### Blinding

Blinding in this type of study is not possible, as knowledge of the group assignment to the participants and providers will be evident. However, to minimize bias in this study, the investigators as well as data analysts will be masked from the respective assignments of the intervention group and TAU group. Blinding will only be terminated after all data collection and analysis have been completed.

### Statistical Methods

#### Primary Outcomes

An intent-to-treat method will be used to conduct the primary analyses. By comparing the baseline and demographic variables between groups, the randomization success will be determined. With a random intercept for a cluster of individuals, generalized linear mixed effects models (GLMMs) will be used [58] to assess the effects of MBCT on depression and psychiatric distress symptoms (hypothesis 1). Week 8 will be the primary end point for this assessment. The covariates measured at TI that are relevant but not balanced between group 1 and group 2 will be incorporated into all models. Additionally, all analysis models will include clinical site level as a fixed effect. GLMMs will be used to test the association between MBCT psychiatric distress (hypothesis 2), in addition to gains sustained at an 8-week follow-up (hypothesis 3).

#### Secondary Outcomes, Moderators, and Mediators

GLMMs will be used to assess the association between MBCT and the secondary outcomes of perceived stress and mindfulness skills (hypothesis 4). Potential moderators of the effect of MBCT, namely treatment history, traumatic life events history, baseline depression, and their interaction with MBCT, will be looked at in their own GLMMs. Using regression coefficients, the role of moderators that these variables may play will be assessed.

Using the PROCESS macro [59] in SAS (SAS Institute), the mediators, extent of mindfulness self-practice, and executive functioning skills (hypothesis 5), will be examined via the product of the coefficient method. This will be limited to the first phase of this study lasting 8 weeks. Further, 2 separate GLMMs will be used to assess how the primary and secondary outcomes may be indirectly influenced by MBCT. By regressing the mediator and outcome, respectively, on MBCT, and controlling for the mediators as well as applicable covariates, the indirect effects of MBCT on outcomes will be examined. Parallel multiple mediator models in addition to serial multiple mediator models will be used to conduct sensitivity analysis. To examine if MBCT effects will be sustained through 8 weeks postintervention (hypothesis 3), linear mixed-effects models will be used with main effects consisting of treatment group, time, group × time interaction, and nested random effects for subjects and cluster. A regression parameter associated with group × time interaction at 0 will be the null hypothesis. This acts as an equivalent for assessing the carryover effect [60]. If rejected, there is support that the effects of the intervention remain after 8 weeks have elapsed. An imputation method will be used to address any data that are missing.

### Potential Bias

It is possible that participants who choose to participate in this study could be more motivated than those who choose not to participate. Participants who are lost to follow-up after randomization could also affect the comparability of the groups.

### Ethical Considerations

This study was approved by the Institutional Review Boards (IRBs) at Greenville Health System and Clemson University (1852922; Pro00092709). All participants will provide written informed consent before participating in this study. This will be collected and safely stored by one of the clinical data collection team members.

### Dissemination

The IRB at Clemson University and Prisma Healthcare System have approved this study. Some of the topics we will discuss are personal and may be upsetting to some participants. If participants experience distress and need treatment, a licensed clinical psychologist or psychiatrist will be contacted immediately. If required, we will provide a list of referral sources for participants who need additional treatment. In the event of physical injury, we will follow emergency procedures for the facility from which the participants are recruited. If an adverse event occurs, a member of the research team will reach out to the affected participant within 24 hours. Subsequently, a meeting will be convened by the research team to discuss the cause of the adverse event and if any modification to this study needs to be made. If any modification needs to be made to the trial protocol, a formal amendment will be submitted to the IRB for approval.

### Data Monitoring Plan

By the National Institutes of Health Office of Human Research Protection, the research team has constructed the Data and Safety Monitoring Plan, safeguarding the well-being and privacy of study participants. Recruitment and safety data will be regularly reviewed by the monitoring board comprised of members from Prisma Health System and Clemson University. Using a personal code that is only accessible by the PI, each participant’s demographic data, and outcome measures will be linked and stored for data analysis. No other identifying data relating to the participant will be linked to these codes. If there are data that are missing, these will also be a component of the monitoring plan for statistical analyses. Every quarter, the board will convene to assess any adverse effects, and on an annual basis, the board will discuss the audition of trial conduct. If the trial needs to be halted, and after consulting the data monitoring committee, the PI will make the final decision to stop all trial proceedings.

### Data Collection and Management

The clinical data collection team will receive training from a licensed clinical psychologist on appropriately gathering data from the PHQ-9 [42] forms to ensure the validity and reliability of trial data. All data collected will be stored electronically and encrypted to ensure confidentiality. This will be done at the clinical facility at which the data are collected. The PI will have full access to the trial data set. To promote the retention of participants, intervention facilitators will encourage continued participation in MBCT sessions, providing the stage to voice any concerns that may have arisen. If concerns arise regarding participation, a member of the research team will reach out to that participant to encourage them to remain.

### Data Reporting Guidelines

The SPIRIT (Standard Protocol Items: Recommendations for Interventional Trials) reporting guidelines [61] were used in the editing of this paper.

## Results

This study began screening and recruitment in December 2022. Data collection from the first cohort occurred in January 2023. As of November 2023, a total of 30 participants have been enrolled out of 224 who received screening. Data analysis is expected to begin in December 2023, with an approximate publication of results by June 2024. IRB approval took place on September 11, 2019.

## Discussion

### Principal Findings

A core aim of this study is to supply the field of research concerning the effect of MBIs on depressive symptoms. To achieve this, we will first address the feasibility and efficacy of delivering MBCT to adults with depressive symptoms via a model delivered via the web. Our unique method of implementation will be tested via a randomized controlled trial with objective markers of depressive symptoms, an adequate overall sample size, and the assessment of adherence and feasibility. Additionally, we will be able to continue postintervention follow-up for 8 weeks, adding to the literature on whether gains can be sustained over time. It is important to assess the capability of MBIs to exert similar effects via web-based formats, as the ability to deliver web-based interventions improves accessibility and affordability of treatment. As such, these interventions may have a broader reach and be used in complement to formal mental health treatment.

Next, we will contribute to the evidence base regarding MBCT’s efficacy in reducing depression symptoms and psychiatric distress. Although pharmacotherapy is widely used and upholds its role in the management of depression, it can be accompanied by medication side effects, delayed onset of therapeutic effects, a lessened impact on milder forms of depression, and an inability to show efficacy in treatment-resistant depression [62]. Furthermore, improper discontinuation of antidepressive medications runs the risk of medicine withdrawal symptoms, and even in the presence of proper medication cessation, there may be a risk of rebound or relapse in depression after discontinuation of antidepressants [63]. Consequently, more evidence supporting MBCT’s role as an adjunct or primary therapy in depression management will be imperative for both clinicians and patients.

We also hope to examine the impact of MBCT on secondary outcomes, including perceived stress and facets of mindfulness. There is increasing evidence in support of perceived stress being positively correlated with the risk of developing depression [64,65]. This may be due to increased activity in the superior frontal gyrus which plays an important role in emotional regulation and cognitive control, with higher spontaneous activity in this region being possibly associated with psychopathologies such as MDD, posttraumatic stress disorder, and social anxiety disorder [66]. If MBCT has a positive impact on perceived stress, this study will add to the understanding of the causal pathway between MBCT, perceived stress, and depressive symptoms. Similarly, given that facets of mindfulness are inversely correlated with components of depression [67], this study can add to the evidence supporting the role that MBCT may play on that causal pathway also.

Through measurement of the extent of mindfulness self-practice and executive function, we hope to better understand the causal pathways between MBCT and depression symptoms. The positive effects of mindfulness practice have been purported to follow a dose-dependent pattern. This may be due to increased functional connectivity within various brain regions [68]. As such, it would be important to assess the role of mindfulness self-practice outside of the dedicated intervention time and its effects on depression. Finally, executive functioning has been shown to have an important influence on emotional regulation abilities [69,70]. The application of MBIs has been shown to be a viable means of improving certain aspects of executive function [71]. Thus, examining executive functioning could provide important insight into MBCT’s ability to influence emotional regulation and depression symptoms.

We will also be able to look at moderators and correlates of intervention outcomes, such as intervention adherence and mental health history, to determine factors that could improve or detract from intervention success.

Overall, this study will generate valuable insights elucidating the possibility of future studies with larger scopes and more elaborate study designs. These may consist of multiple evidence-based comparisons and longer postintervention follow-ups.

### Trial Status

Recruitment for the trial began in December 2022 and will last approximately until the sample size of 128 individuals is reached or 4 years have elapsed (September 2026).

## Abbreviations

DSM-5: Diagnostic and Statistical Manual of Mental Disorders (Fifth Edition)
GLMM: generalized linear mixed effects model
ICD-11: International Classification of Diseases, 11th Revision
IRB: institutional review board
MBCT: mindfulness-based cognitive therapy
MBI: mindfulness-based intervention
MDD: major depressive disorder
PHQ-9: Patient Health Questionnaire-9
PI: principal investigator
SPIRIT: Standard Protocol Items: Recommendations for Interventional Trials
TAU: treatment as usual

## Data Availability

Data gathered from this trial will be available to the public and research community. The research team will convene before the end of the trial to select the appropriate method to disseminate the data collected.
